# Bicarbonate defective *CFTR* variants increase risk for chronic pancreatitis: A meta-analysis

**DOI:** 10.1371/journal.pone.0276397

**Published:** 2022-10-20

**Authors:** Gergő Berke, Noémi Gede, Letícia Szadai, Klementina Ocskay, Péter Hegyi, Miklós Sahin-Tóth, Eszter Hegyi

**Affiliations:** 1 Institute for Translational Medicine, Medical School, University of Pécs, Pécs, Hungary; 2 Department of Dermatology and Allergology, University of Szeged, Szeged, Hungary; 3 Centre for Translational Medicine, Semmelweis University, Budapest, Hungary; 4 Division of Pancreatic Diseases, Heart and Vascular Centre, Semmelweis University, Budapest, Hungary; 5 Department of Surgery, University of California, Los Angeles, Los Angeles, California, United States of America; Heidelberg University, GERMANY

## Abstract

**Introduction:**

Cystic fibrosis transmembrane conductance regulator (CFTR) plays a central role in pancreatic ductal fluid secretion by mediating Cl^-^ and HCO_3_^-^ ion transport across the apical membrane. Severe *CFTR* mutations that diminish chloride conductance cause cystic fibrosis (CF) if both alleles are affected, whereas heterozygous carrier status increases risk for chronic pancreatitis (CP). It has been proposed that a subset of *CFTR* variants characterized by a selective bicarbonate conductance defect (*CFTR*^BD^) may be associated with CP but not CF. However, a rigorous genetic analysis of the presumed association has been lacking.

**Aims:**

To investigate the role of heterozygous *CFTR*^BD^ variants in CP by meta-analysis of published case-control studies.

**Materials and methods:**

A systematic search was conducted in the MEDLINE, Embase, Scopus, and CENTRAL databases for published studies that reported the *CFTR*^BD^ variants p.R74Q, p.R75Q, p.R117H, p.R170H, p.L967S, p.L997F, p.D1152H, p.S1235R, and p.D1270N in CP patients and controls.

**Results:**

Twenty-two studies were eligible for quantitative synthesis. Combined analysis of the 9 *CFTR*^BD^ variants indicated enrichment in CP patients versus controls (OR = 2.31, 95% CI = 1.17–4.56). Individual analysis of *CFTR*^BD^ variants revealed no association of p.R75Q with CP (OR = 1.12, 95% CI = 0.89–1.40), whereas variants p.R117H and p.L967S were significantly overrepresented in cases relative to controls (OR = 3.16, 95% CI = 1.94–5.14, and OR = 3.88, 95% CI = 1.32–11.47, respectively). The remaining 6 low-frequency variants gave inconclusive results when analyzed individually, however, their pooled analysis indicated association with CP (OR = 2.08, 95% CI = 1.38–3.13).

**Conclusion:**

Heterozygous *CFTR*^BD^ variants, with the exception of p.R75Q, increase CP risk about 2-4-fold.

## Introduction

Chronic pancreatitis (CP) is a progressive inflammatory disorder of the pancreas, which develops due to an interplay between environmental and genetic risk factors [1]. Research on the identification of the underlying genetic basis has been rapidly evolving; there are at least a dozen genes reported to date that may contribute to CP risk. Based on their function, these susceptibility genes and their alterations can be categorized into three distinct pathophysiological pathways [2]. Genetic variants in the so-called trypsin-dependent pathway alter premature intrapancreatic trypsinogen activation, and include the serine protease 1 and 2 (*PRSS1*, *PRSS2*) genes encoding human cationic and anionic trypsinogen, the serine protease inhibitor Kazal type 1 (*SPINK1*) gene, the chymotrypsinogen C (*CTRC*) gene, and an inversion at the chymotrypsinogen B1-B2 (*CTRB1-CTRB2*) locus [3]. Variants in the misfolding-dependent pathological pathway induce digestive enzyme misfolding and endoplasmic reticulum stress. Certain variants in *PRSS1*, *CTRC*, carboxypeptidase A1 (*CPA1*), and carboxyl ester lipase (*CEL*) belong to this group [4]. Finally, variants in the ductal pathway of CP risk affect genes encoding different channels expressed predominantly in the pancreatic ductal epithelial cells. These include alterations in the cystic fibrosis transmembrane conductance regulator (*CFTR*) gene, the transient receptor potential cation channel subfamily V member 6 (*TRPV6)* gene, and the claudin 2—MORC family CW-type zinc finger 4 (*CLDN2-MORC4*) locus [2]. More recently, protease-sensitive pancreatic lipase (*PNLIP*) variants, and the loss-of-function c.129+1G>A chymotrypsin like elastase 3B (*CELA3B*) variant have been reported to increase CP risk. The pathomechanism by which these variants promote the development of CP remains to be elucidated [5, 6].

CFTR is a chloride (Cl^-^)/bicarbonate (HCO_3_^-^) ion channel expressed in the secretory epithelia of airways, gastrointestinal tract, pancreas, reproductive organs, and exocrine glands [7]. In the pancreas it has a dual function; CFTR-mediated HCO_3_^-^ secretion drives the transepithelial fluid secretion in pancreatic ducts while maintaining the characteristic alkaline pH of the pancreatic juice. Mutations in *CFTR* that diminish the ion channel function and lead to impaired epithelial fluid transport cause cystic fibrosis (CF), the most common autosomal recessive disorder among European populations. When both *CFTR* alleles harbor severe loss-of-function mutations, CF with pancreatic insufficiency develops [8]. A severe mutation on one *CFTR* allele and a milder mutation on the other allele with some residual CFTR function may result in CF with pancreatic sufficiency or in CFTR-related disorders such as CP. Heterozygous carriers of *CFTR* mutations do not develop CF but exhibit increased risk for CP [8].

The Whitcomb laboratory proposed that certain *CFTR* mutations that are not associated with CF may be risk factors for CP by preferentially lowering the HCO_3_^-^ conductance and permeability of the CFTR channel (bicarbonate defective *CFTR* variants; *CFTR*^BD^) [9, 10]. Genetic and functional assays identified 9 such *CFTR*^BD^ variants (p.R74Q, p.R75Q, p.R117H, p.R170H, p.L967S, p.L997F, p.D1152H, p.S1235R, and p.D1270N). Subsequent studies, however, failed to replicate the association of the relatively frequent variant p.R75Q with CP, raising doubt about the clinical relevance of the *CFTR*^BD^ variants [11, 12]. To resolve this controversy, here we investigated the role of *CFTR*^BD^ variants in CP by a meta-analytical approach.

## Methods

### Search strategy

Two authors independently performed a systematic search on June 7, 2022, in four databases (MEDLINE via Pubmed, Embase, Scopus, and Cochrane Library) using the following search key: ((CFTR related disorders) OR (CFTR RD) OR pancreatitis)) AND ((CFTR OR (cystic fibrosis transmembrane conductance regulator) AND (mutation* OR variant* OR polymorphism*)). To reduce the number of results in Scopus, the search was conducted within the ‘article title, abstract, keywords’ fields. Language or date restrictions were not applied. Citing (using MEDLINE via Pubmed and Google Scholar) and cited reference searches were conducted on June 23, 2022.

### Protocol registration

The present work is reported in accordance with the Preferred Reporting Items for Systematic Reviews and Meta-Analyses (PRISMA) Statement (S1 Checklist) [13]. The protocol of the meta-analysis was registered in advance in the PROSPERO database under the registration number CRD42020163218.

### Selection criteria and data extraction

The study selection process was completed by two authors using a reference management program (Endnote X7.5; Clarivate Analytics, Philadelphia, PA). Genetic association case-control studies with adequately defined CP patients [14] and controls investigating some or all previously reported *CFTR*^BD^ variants (p.R74Q, p.R75Q, p.R117H, p.R170H, p.L967S, p.L997F, p.D1152H, p.S1235R, and p.D1270N) were included. Studies (i) analyzing autoimmune, hereditary, or familial chronic pancreatitis; (ii) with overlapping cohorts; and (iii) without proper allele frequency or genotype distribution data were excluded.

Eligible original studies were subjected to data collection onto a pre-defined Excel sheet by two authors independently. The following data were extracted: first author, publication year, cohort ethnicity, range and mean age of participants, etiology of CP, number of cases and controls, genotyping method, allele frequencies of *CFTR*^BD^ variants, and *SPINK1* p.N34S carrier status. In some cases, allele frequencies were calculated from the reported genotype distribution.

### Quality assessment

Quality of the included studies was assessed by two authors independently using the modified version of the Newcastle-Ottawa Scale (NOS) and by calculating the Hardy-Weinberg Equilibrium with the χ^2^ test (S1 Table).

Discrepancies during search, selection, data extraction, and quality evaluation between authors were resolved by the corresponding author or by mutual agreement.

### Statistical analysis

Combined or individual effects of *CFTR*^*BD*^ variants were assessed by calculating pooled odds ratios (OR) with 95% confidence intervals (CI) using the random-effects model with Der-Simonian Laird estimation. Results were displayed on forest plots. To determine the cumulative effect of *CFTR*^*BD*^ variants, studies investigating all nine variants were included. Heterogeneity between studies was investigated with the I^2^ (p≥0.1) and χ^2^tests, interpretation of results was based on the Cochrane Handbook for Systematic Reviews of Interventions version 6.3 [15]. Sensitivity analysis was carried out by repeating the quantitative synthesis while leaving out one study at a time (leave-one-out method). Where the number of included studies allowed, funnel plots were generated to rule out publication bias and the small study effect was estimated by Egger’s test. The effect of compound heterozygosity for *CFTR*^BD^ and *SPINK1* p.N34S variants was assessed by Fisher’s exact test. Statistical analyses were performed with the Stata 15 (Stata Corp) program.

## Results

**Fig 1:**
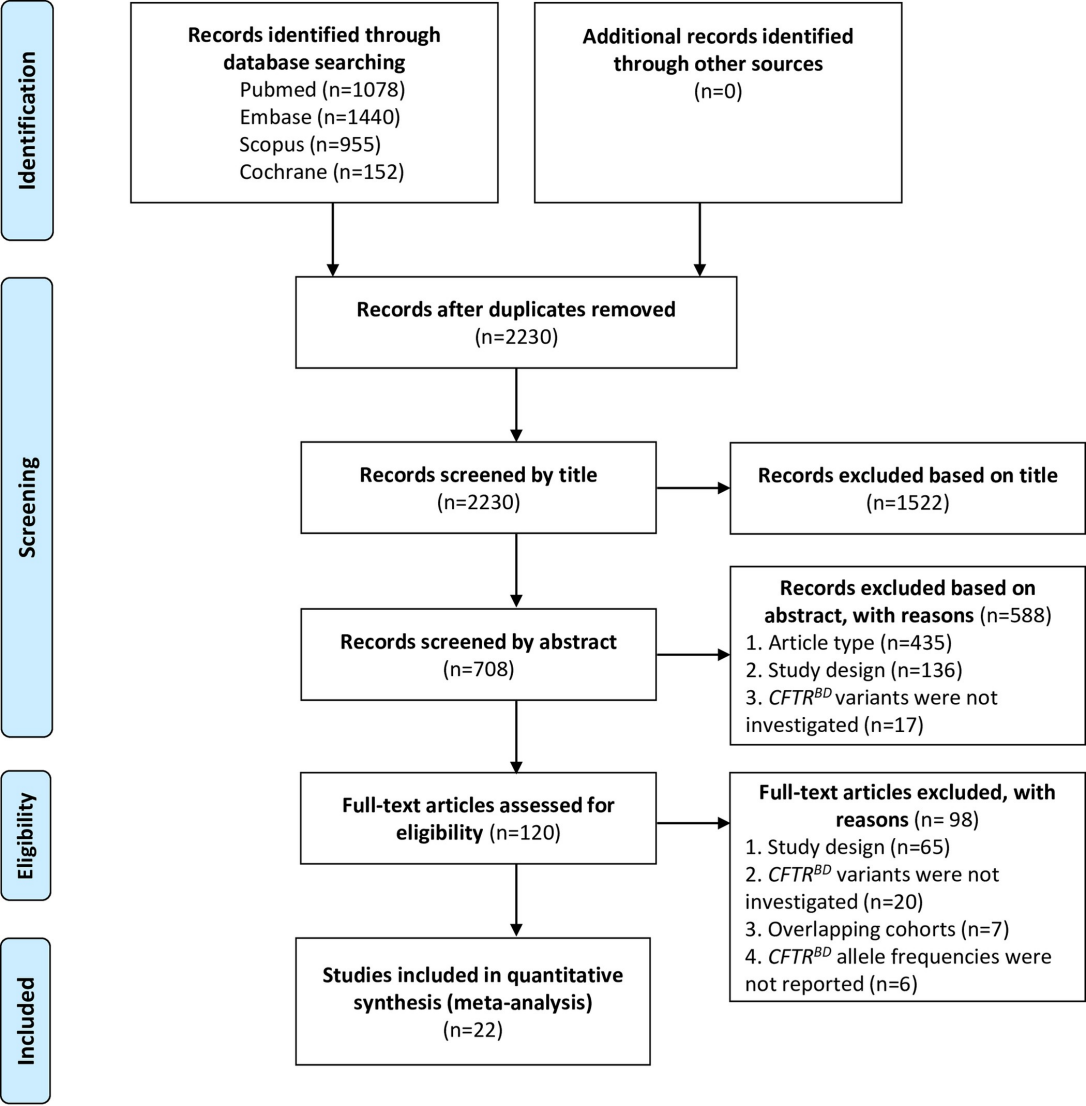
PRISMA flow diagram showing the systematic search and selection process.

The comprehensive systematic search and selection process identified 22 case-control studies that reported on some or all of the 9 *CFTR*^BD^ variants and met the inclusion criteria for quantitative synthesis (Fig 1) [9, 11, 12, 16–34]. We noted significant geographic/ethnic differences in the distribution of the *CFTR*^BD^ variants. In the cohorts of European origin or ancestry, the overall allele frequency of all *CFTR*^BD^ variants was 6.1% (174/2862) in patients and 3.6% (130/3592) in controls, whereas *CFTR*^BD^ variants were nearly absent in the Indian and East-Asian cohorts (S2 and S3 Tables). Although *CFTR*^BD^ variants were relatively common in an African American cohort, there was no difference between their allelic distribution in patients (10/464, 2.2%) and controls (10/476, 2.1%, OR = 1.03; 95% CI 0.42–2.49; p = 0.95). To avoid the potentially confounding effect of these geographic/ethnic disparities, we focused our analysis on cohorts of European origin. All *CFTR*^BD^ variants were reported in the heterozygous state with the sole exception of the p.R75Q variant, which was also detected in the homozygous state in 3 patients and 1 control. Hence, our analyses considered allele frequencies only.

### Association analysis

**Fig 2:**
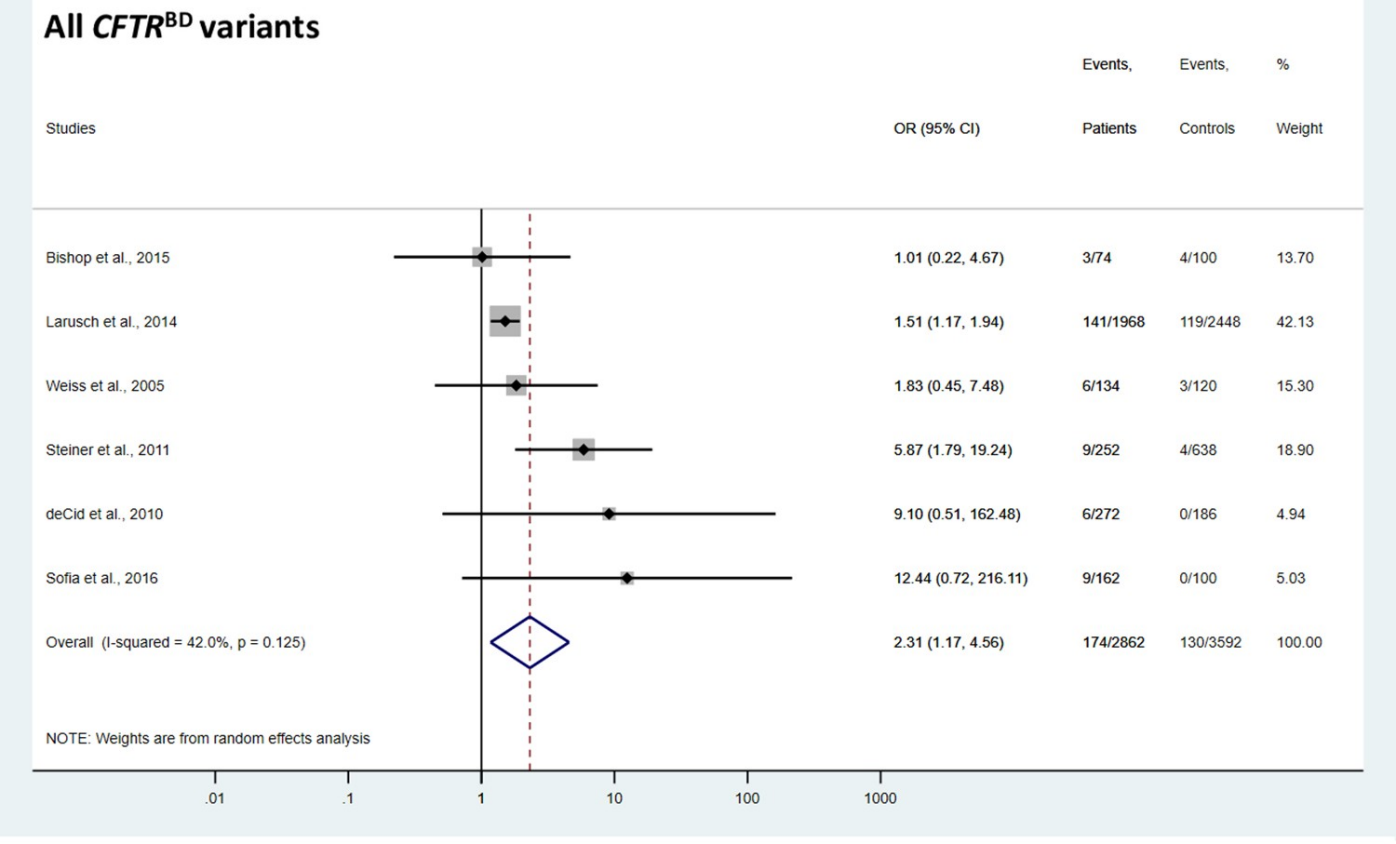
Forest plot showing cumulative odds ratios for chronic pancreatitis risk in carriers of *CFTR*^BD^ variants. OR, odds ratio; CI, confidence interval.

**Fig 3:**
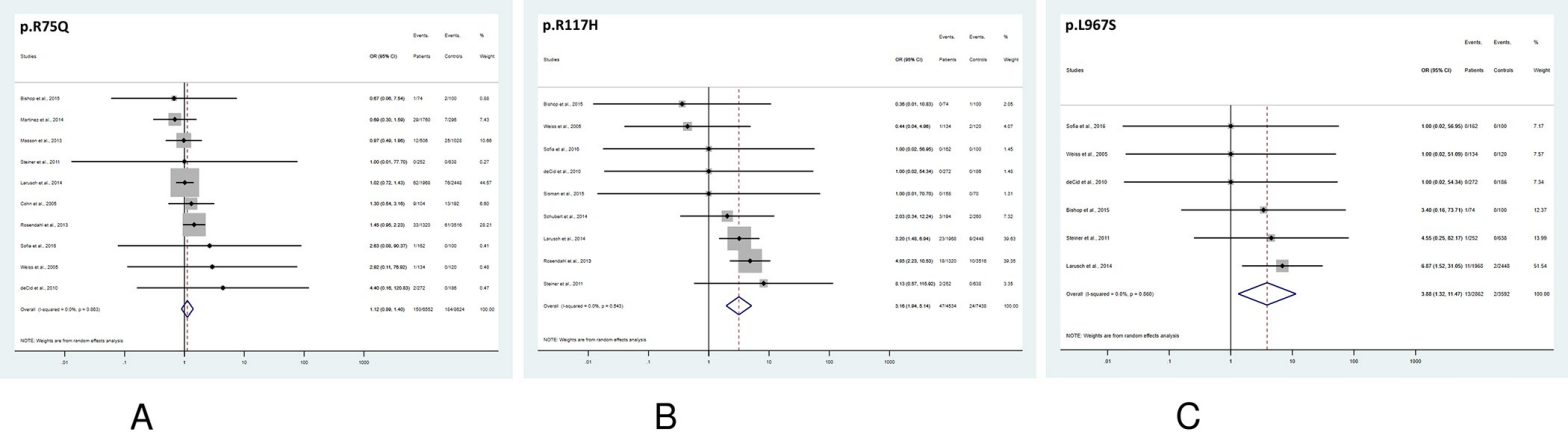
Forest plot showing odds ratios for chronic pancreatitis risk in subjects with *CFTR* variants. **A,** p.R75Q; **B,** p.R117H; **C,** p.L967S. OR, odds ratio; CI, confidence interval.

**Fig 4:**
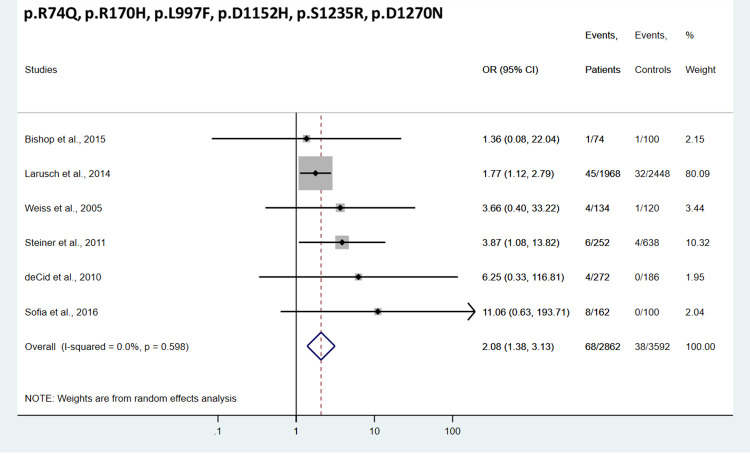
Forest plot showing cumulative odds ratios for chronic pancreatitis risk in subjects carrying *CFTR* variants p.R74Q, p.R170H, p.L997S, p.D1152H, p.S1235R, and p.D1270N. OR, odds ratio; CI, confidence interval.

The aggregate analysis of the 9 *CFTR*^BD^ variants (p.R74Q, p.R75Q, p.R117H, p.R170H, p.L967S, p.L997F, p.D1152H, p.S1235R, and p.D1270N) showed significant association with CP (OR = 2.31, 95% CI = 1.17–4.56) (Fig 2). When analyzed individually, conclusive results were obtained for 3 *CFTR*^BD^ variants, p.R75Q, p.R117H, and p.L967S. The most common variant p.R75Q showed no association with CP (OR = 1.12, 95% CI = 0.89–1.40) (Fig 3A). In contrast, variants p.R117H and p.L967S were significantly overrepresented in CP cases relative to controls (OR = 3.16, 95% CI = 1.94–5.14, and OR = 3.88, 95% CI = 1.32–11.47, respectively) (Fig 3B and 3C). Individual analysis of the remaining 6 *CFTR*^BD^ variants (p.R74Q, p.R170H, p.L997F, p.D1152H, p.S1235R, and p.D1270N) gave inconclusive results due to their low frequency in the studied cohorts (S1–S4 Figs). However, a pooled analysis of these 6 variants showed significant enrichment in CP cases versus controls (OR = 2.08, 95% CI = 1.38–3.13) (Fig 4).

No substantial heterogeneity was observed among studies. Sensitivity analysis (leave-one-out method) revealed a significant impact of the largest cohort study conducted by Larusch et al. (2014) on the summary OR values in case of three variants; omitting this study resulted in loss of significance in case of the p.L967S and p.S1235R variants, while the calculated risk became significant in case of the p.L997F variant.

### Quality assessment and publication bias

Assessment of the Hardy-Weinberg equilibrium in control subjects for the individual *CFTR*^BD^ variants revealed no deviations in the included studies (S1 Table). Based on the modified Newcastle-Ottawa Scale, all studies met the excellent-quality criteria (S1 Table). Funnel plots were generated for the p.R75Q, p.R117H, and p.D1152H variants (S4 Fig).

## Discussion

The pathogenic significance of *CFTR* mutations has been established not only in the development of CF, but also in CFTR-related disorders such as congenital absence of the vas deferens, chronic sinopulmonary disorders, and CP. Observations that heterozygous and compound heterozygous *CFTR* mutations are linked with CP were first reported in the late 1990s [35, 36]. Large cohort analyses confirmed the initial findings and determined an increased risk for heterozygous carriers of the severe p.F508del mutation (OR~2.5), and the mild p.R117H mutation (OR~4) [9, 11, 12, 18–20, 37]. Compound heterozygous carriers of a severe and a mild *CFTR* variants are at increased risk of developing CP and such constellations can be considered causative [8, 12]. It has been proposed that a subset of 9 *CFTR* variants somewhat selectively reduces the permeability of CFTR to HCO^3-^ and thereby increases risk for CP [9, 10]. According to the CFTR2 database, when combined with a CF-causing allele, these *CFTR*^BD^ variants do not cause CF or have variable clinical effects [38].

The aim of the present study was to determine the risk of CP in heterozygous *CFTR*^BD^ carriers using meta-analysis of published case-control studies. First, we investigated the association of CP with all 9 *CFTR*^BD^ variants combined, based on the assumption that these variants act via the same pathomechanism. We found that *CFTR*^BD^ variants increased CP risk by about 2.3-fold, as estimated by the odds ratio. Next, individual analysis of the 9 *CFTR*^BD^ variants was performed. Two variants, p.R117H and p.L967S, showed significant association with CP with moderate effect sizes (OR~3.2 and 3.9, respectively). The CFTR2 database reports the p.R117H variant as a mild CF-associated mutation with variable clinical consequences influenced by the length of the poly-T tract in intron 8. The variant acts as CF-causing when found *in cis* with the T5 tract [38]. When combined with a T7 tract *in cis* and a CF-causing variant *in trans*, some subjects develop CF while others do not. Notwithstanding the poly-T tract length of p.R117H carriers, CF patients are likely to remain pancreatic sufficient. With respect to CP, it seems that p.R117H increases disease risk regardless of the poly-T tract status [9, 11]. Unfortunately, due to the limited data on the intron 8 poly-T tract in the studies included in our meta-analysis, we could not investigate this relationship further. Mechanistically, the p.R117H is an outlier among the *CFTR*^*BD*^ variants, as it significantly reduces Cl^-^ transport, while the other 8 variants have minimal or no impact on this CFTR function (see Fig 1B in [9]). A recent study demonstrated that mutation p.R117H impairs channel gating due to the loss of a hydrogen bond between the side chain of Arg117 and the backbone carbonyl group of Glu1124 [39].

Association of the p.L967S variant with CP was mainly driven by a single study with the largest cohort [9], as determined by the sensitivity analysis. Since the variant was found only once in two other studies each, the overall confidence regarding the effect size of the demonstrated disease association remains tempered. The CFTR2 database indicates that the p.L967S variant has varying clinical consequences. When combined with another CF-causing variant, it may or may not cause CF. Patients with CF who have this variant are likely to be pancreatic sufficient [38].

In contrast to the p.R117H and p.L967S variants, variant p.R75Q was not associated with CP. According to the CFTR2 database, this variant does not cause CF when combined with a CF-causing variant [38]. Earlier studies suggested that p.R75Q elevates CP risk in subjects transheterozygous for the *SPINK1* p.N34S mutation [10, 40]. We were unable to perform a rigorous test of this assumption, because of the very low number of transheterozygotes in controls. We considered comparing the detected and expected number of transheterozygous CP patients, however, ethnic/geographic differences in the carrier frequency of the individual variants makes the prediction of the expected number unreliable. Furthermore, we note that there are no other examples in the genetics of CP when a variant would confer no disease risk but it would act pathogenic when combined with another risk variant. To date, all identified CP-associated genetic variants seem to be independent risk factors whose effects become multiplied in carriers of multiple variants. Finally, there is no known mechanistic link between the SPINK1 and CFTR proteins, which might suggest a direct interaction.

Due to the relatively rare occurrence of the remaining 6 *CFTR*^BD^ variants (p.R74Q, p.R170H, p.L997F, p.D1152H, p.S1235R, and p.D1270N), individual analyses were inconclusive, although all variants showed a trend toward disease association. When we calculated their combined effect size, we found significant association with CP (OR~2.1). Variants p.D1152H and p.D1270N are listed in the CFTR2 database as having a variable clinical effect [38]. CF patients with these variants are likely to be pancreatic sufficient. The database lists p.R170H, p.L997F, and p.S1235R as non-CF causing variants. There is no entry for p.R74Q, however, another variant that affects the same position, p.R74W, is associated with a variable clinical phenotype. Taken together, it appears that this group of heterozygous *CFTR* variants increases CP risk modestly, but the effect sizes of the individual variants cannot be determined with confidence until more data becomes available.

Finally, it is curious to note that genetic effect size correlates poorly with the reported functional defects in the *CFTR*^BD^ variants. Thus, variant p.R75Q, which does not alter CP risk, had a significant impact on HCO_3_^-^ permeability and conductance (see Fig 1E and 1F in [9]). In contrast, variant p.L967S, which showed the highest OR in our analysis, had the smallest impact on ion permeability. We also note that the published functional analysis was performed under somewhat artificial conditions, with CFTR variants as well as WNK1 and SPAK overexpressed in transfected HEK 293T cells. It is possible, even likely, that the *CFTR*^BD^ variants behave somewhat differently in their native environment, which might explain these discrepancies.

Taken together, our meta-analysis confirmed that with the sole exception of p.R75Q, *CFTR*^*BD*^ variants increase the risk for CP by approximately 2-4-fold. The limitation of this meta-analysis is the relatively small cohort size in many of the included studies, which likely precluded detection of some of the rare variants. Furthermore, due to the limited data available, no subgroup analyses regarding CP etiology could be performed.

## Supporting information

S1 Checklist PRISMA 2020 checklist. (PDF)

S1 Fig Forest plot showing odds ratios for chronic pancreatitis risk in carriers of *CFTR* variants. **A,** p.R74Q; **B**, p.R170H. OR, odds ratio; CI, confidence interval.
(PPTX)

S2 Fig Forest plot showing odds ratios for chronic pancreatitis risk in carriers of *CFTR* variants. **A,** p.L997F; **B,** p.D1152H. OR, odds ratio; CI, confidence interval.
(PPTX)

S3 Fig Forest plot showing odds ratios for chronic pancreatitis risk in carriers of *CFTR* variants. **A,** p.S1235R; **B,** p.D1270N. OR, odds ratio; CI, confidence interval.
(PPTX)

S4 Fig Funnel plots evaluating the effect of publication bias. **A,** p.R75Q; **B,** p.R117H; **C,** p.D1152H.
(PPTX)

S1 Table Newcastle-Ottawa Scale (NOS) for quality assessment of the case-control studies selected for meta-analysis. (DOCX)

S2 Table Characteristics of studies included in the meta-analysis. (DOCX)

S3 Table Allele frequency of *CFTR*^*BD*^ variants in studies included in the meta-analysis. (DOCX)
