# Supplementary figures and images for: Bicarbonate defective CFTR variants increase risk for chronic pancreatitis: A meta-analysis
Source: PLoS One. 2022 Oct 20;17(10):e0276397. doi: 10.1371/journal.pone.0276397 (PMC9584382; doi:10.1371/journal.pone.0276397)

## Slide 1
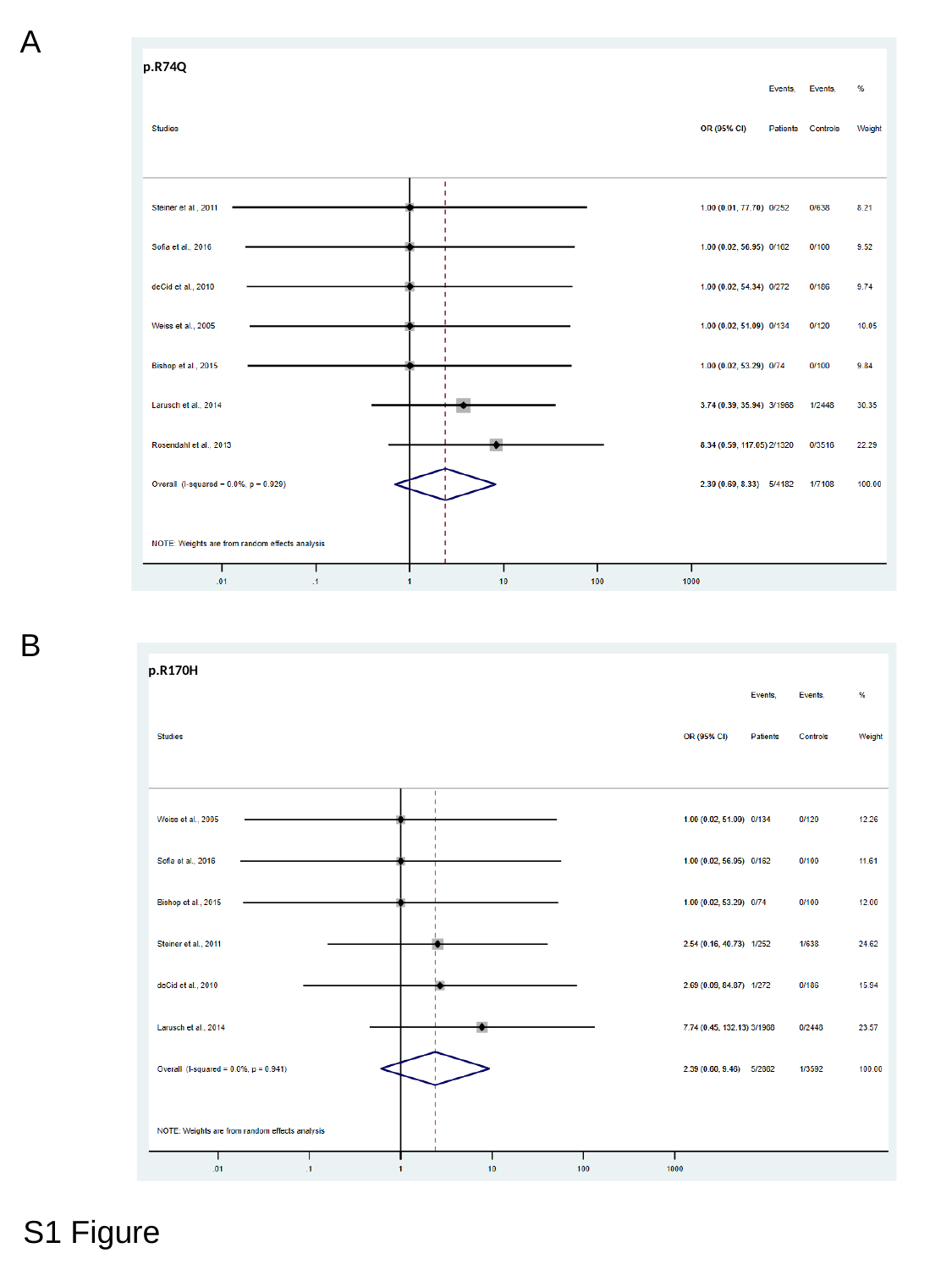

A
p.R74Q
B
p.R170H
S1 Figure

Supplement: S1 Fig — A, p.R74Q; B, p.R170H. OR, odds ratio; CI, confidence interval. (PPTX) [file pone.0276397.s002.pptx]

## Slide 1
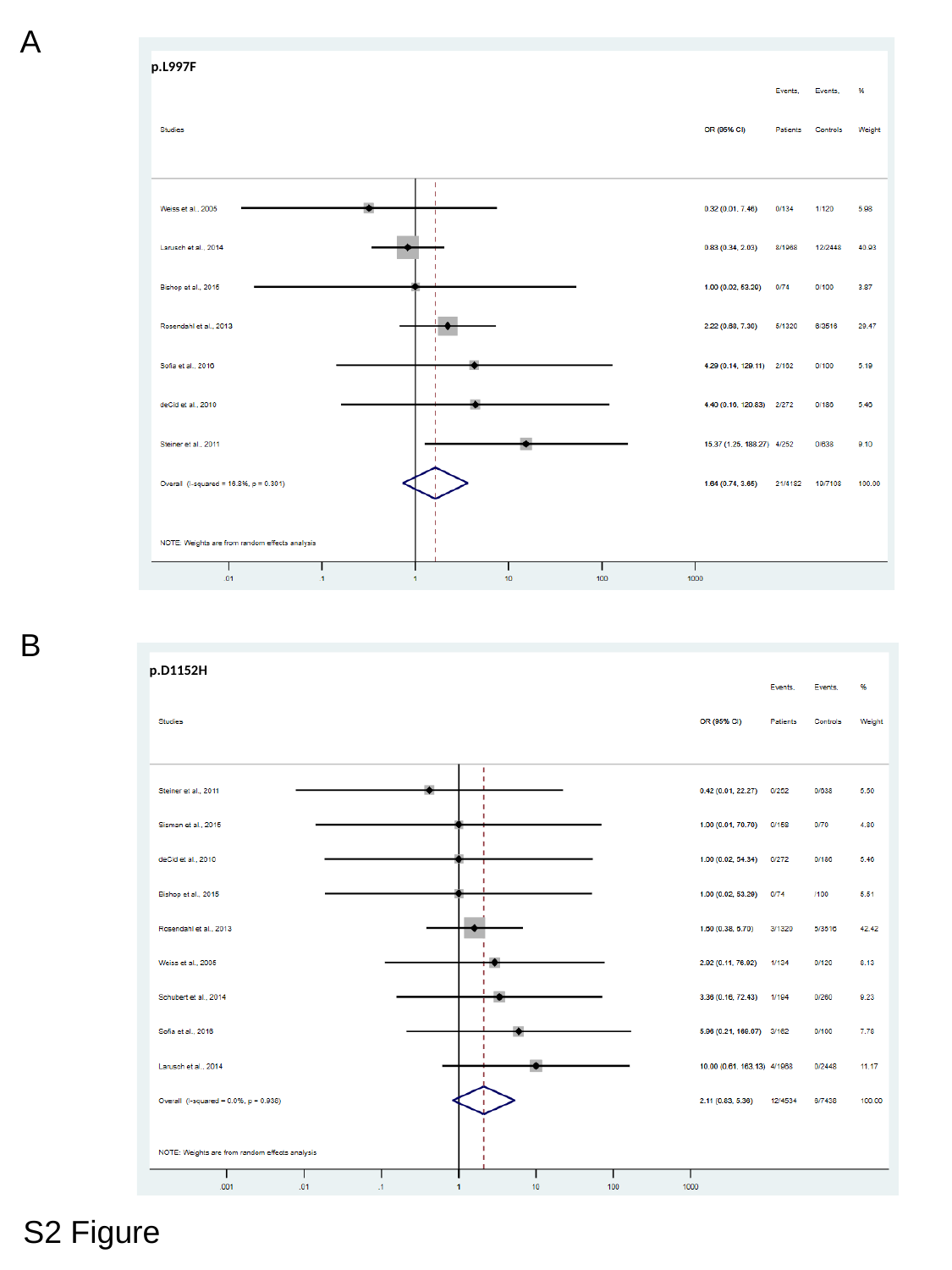

A
p.L997F
B
p.D1152H
S2 Figure

Supplement: S2 Fig — A, p.L997F; B, p.D1152H. OR, odds ratio; CI, confidence interval. (PPTX) [file pone.0276397.s003.pptx]

## Slide 1
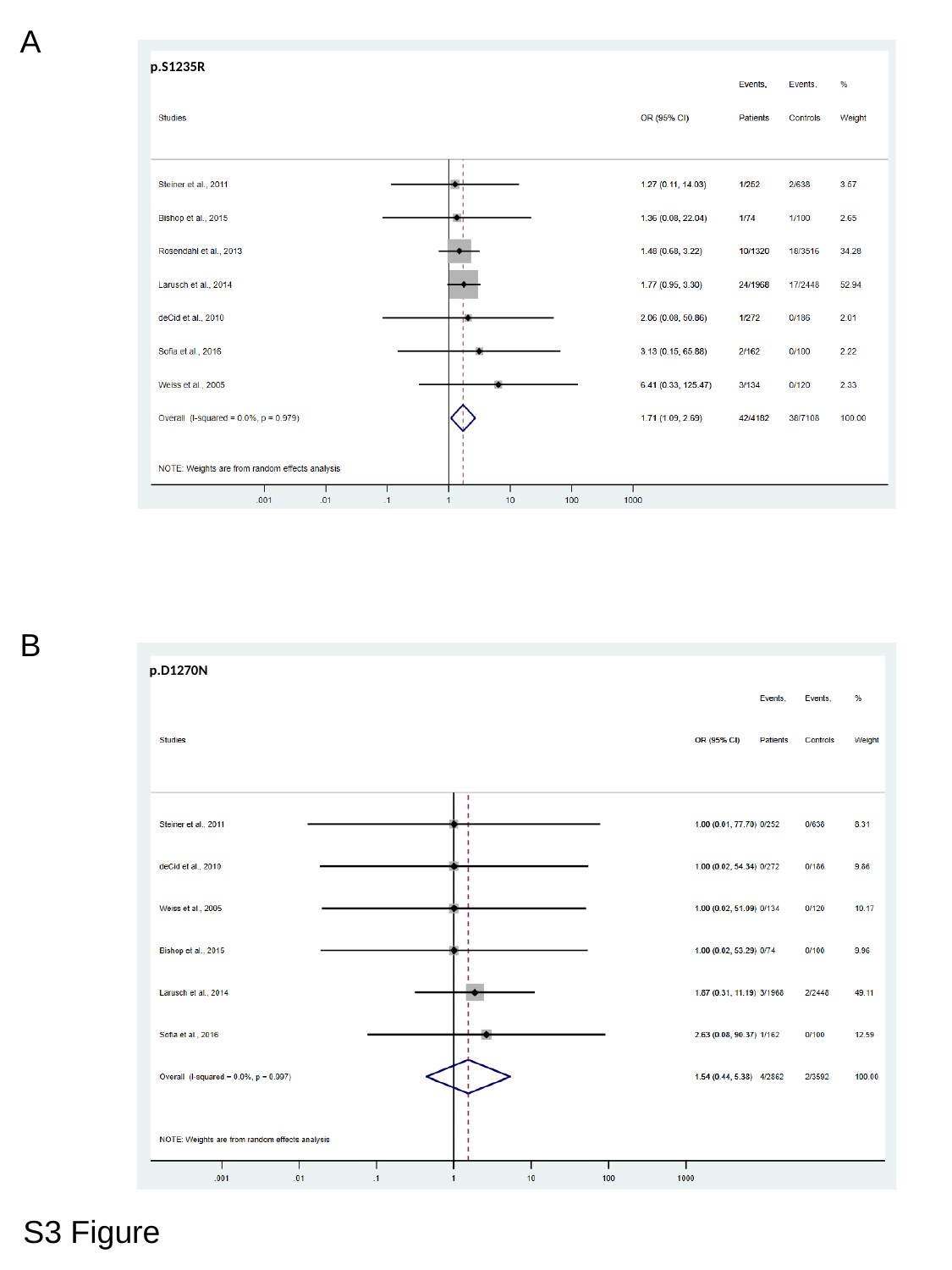

A
p.S1235R
B
p.D1270N
S3 Figure

Supplement: S3 Fig — A, p.S1235R; B, p.D1270N. OR, odds ratio; CI, confidence interval. (PPTX) [file pone.0276397.s004.pptx]

## Slide 1
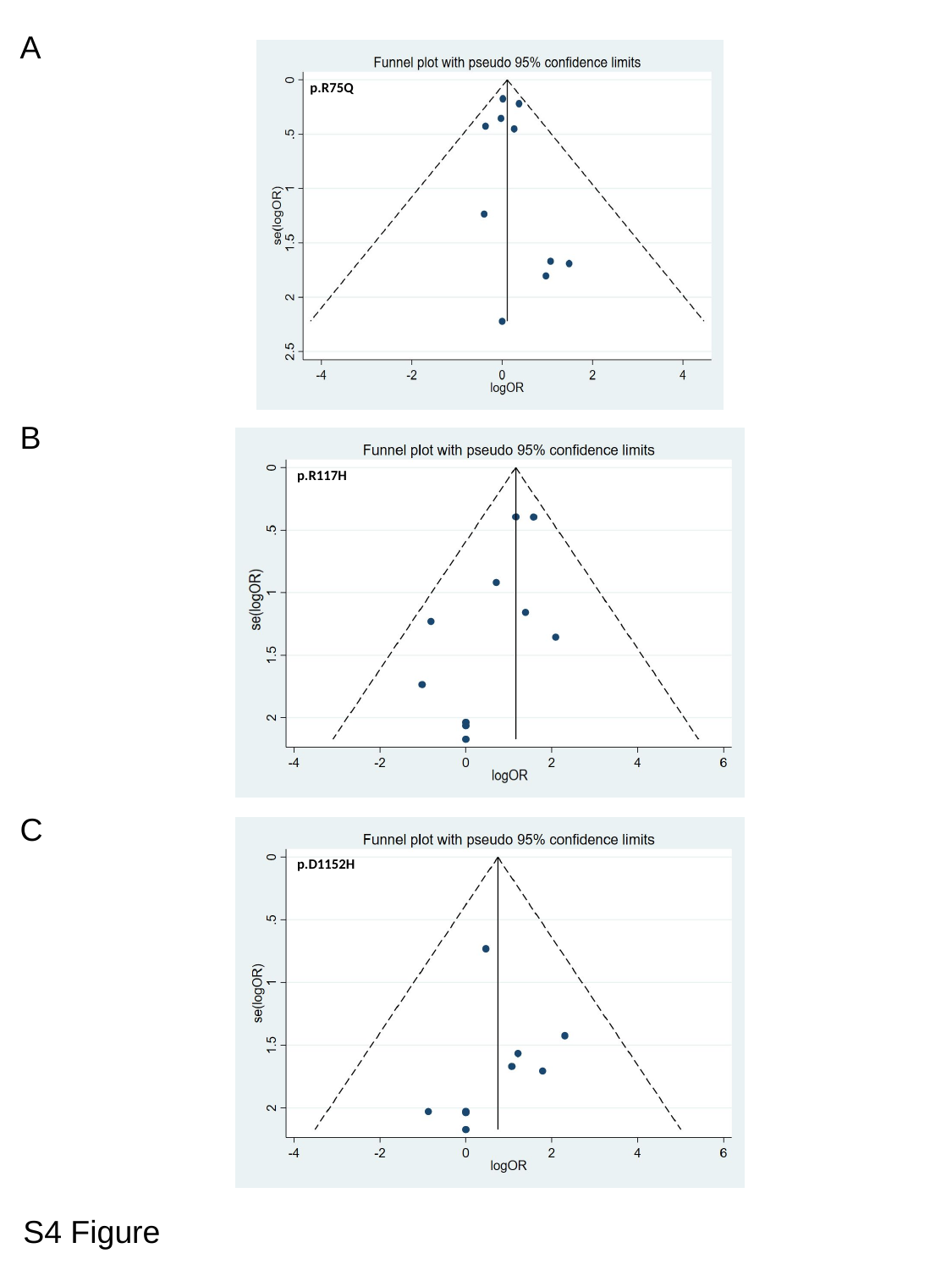

A
p.R75Q
B
p.R117H
C
p.D1152H
S4 Figure

Supplement: S4 Fig — A, p.R75Q; B, p.R117H; C, p.D1152H. (PPTX) [file pone.0276397.s005.pptx]
