# Supplementary material for: Bicarbonate defective CFTR variants increase risk for chronic pancreatitis: A meta-analysis
Source: PLoS One. 2022 Oct 20;17(10):e0276397. doi: 10.1371/journal.pone.0276397 (PMC9584382; doi:10.1371/journal.pone.0276397)
Supplement: S1 Table — (DOCX) [file pone.0276397.s006.docx]

**S1 Table. Hardy-Weinberg Equilibrium and Newcastle-Ottawa Scale (NOS) for quality assessment of the case-control studies selected for meta-analysis.** Quality assessment scale detailed on the website of The Ottawa Hospital Research Institute (<https://www.ohri.ca/programs/clinical_epidemiology/oxford.asp>).

| Study | Selection | | | | Comparability | Exposure | | HWE |
| --- | --- | --- | --- | --- | --- | --- | --- | --- |
|  | CD | REC | SC | DC | CCC | AE | SM |  |
| Lee et al., 2003 | + | + | + | + | + | + | + | + |
| Fujiki et al., 2004 | + | + | + | + | + | + | + | + |
| Bishop et al., 2005 | + | + | + | + | + | + | + | + |
| Cohn et al., 2005 | + | + | + | + | + | + | + | + |
| Weiss et al., 2005 | + | + | + | + | + | + | + | + |
| Chang et al., 2007 | + | + | + | + | + | + | + | + |
| Aoyagi et al., 2009 | + | + | + | + | + | + | + | + |
| de Cid et al., 2010 | + | + | + | + | + | + | - | + |
| Midha et al., 2010 | + | + | + | + | + | + | + | + |
| Steiner et al., 2011 | + | + | + | + | + | + | - | + |
| Rosendahl et al., 2013 | + | + | + | + | + | + | + | + |
| Masson et al., 2013 | + | + | + | + | + | + | - | + |
| Larusch et al., 2014 | + | + | + | + | + | + | - | + |
| Martinez et al., 2014 | + | + | + | + | + | + | + | + |
| Muthuswamy et al., 2014 | + | + | + | + | + | + | + | + |
| Schubert et al., 2014 | + | + | + | + | + | + | + | + |
| Sisman et al., 2015 | + | + | + | + | + | + | + | + |
| Sofia et al., 2016 | + | + | + | + | + | + | + | + |
| Philips et al., 2018 | + | + | + | + | + | + | + | + |
| Zou et al., 2018 | + | + | + | + | + | + | + | + |
| Iso et al., 2019 | + | + | + | + | + | + | + | + |
| Chonchubhair et al., 2020 | + | + | + | + | + | + | + | + |

CD, cases defined adequately; REC, representativeness of the cases; SC, selection of controls; DC, definition of controls; CCC, comparability of cases and controls; AE, ascertainment of exposure; SMA, same method of ascertainment for cases and controls; HWE, Hardy-Weinberg equilibrium in controls.
