# Supplementary material for: Bicarbonate defective CFTR variants increase risk for chronic pancreatitis: A meta-analysis
Source: PLoS One. 2022 Oct 20;17(10):e0276397. doi: 10.1371/journal.pone.0276397 (PMC9584382; doi:10.1371/journal.pone.0276397)
Supplement: S2 Table — (DOCX) [file pone.0276397.s007.docx]

**S2 Table. Characteristics of studies included in the meta-analysis.**

| Study | Demographic characteristics | | | Investigated  variants | Cohort | MAF of investigated *CFTR*^BD^ variants | Genotyping method |
| --- | --- | --- | --- | --- | --- | --- | --- |
|  | Ethnicity | Age (mean ±SD/range) | Etiology |  |  |  |  |
| Lee et al., 2003 | Korean | NA/NA | NACP | All (9) | CP | 0/56 (0%) | TDGS and Sanger sequencing |
|  |  |  |  |  | Controls | 1/234 (0.4%) |  |
| Fujiki et al., 2004 | Japanese | 61/27–82 (ACP)  57/13–85 (ICP) | ACP  ICP | p.R117H | CP | 0/130 (0%) | Elucigene CF20 kit |
|  |  |  |  |  | Controls | 0/324 (0%) |  |
| Bishop et al., 2005 | North American | NA/9-71 | ICP | All (9) | CP | 3/74 (4.1%) | PCR-based multiplex, heteroduplex analysis on the MDE gel matrix |
|  |  |  |  |  | Controls | 4/100 (4%) |  |
| Cohn et al., 2005 | European | NA/NA | ICP | p.R75Q | CP | 9/104 (8.7%) | DOVAM-S |
|  |  |  |  |  | Controls | 13/192 (6.8%) |  |
| Weiss et al., 2005 | German | NA/3–68 | ICP | All (9) | CP | 6/134 (4.5%) | Sanger sequencing |
|  |  |  |  |  | Controls | 3/120 (2.5%) |  |
| Chang et al., 2007 | Taiwanese | NA/NA | ICP | All (9) | CP | 0/156 (0%) | DHPLC and Sanger sequencing |
|  |  |  |  |  | Controls | 0/400 (0%) |  |
| Aoyagi et al., 2009 | Japanese | 53.2±12.2/ NA (ACP)  42.6±10.2/ NA (ICP) | ACP  ICP | All (9) | CP | 0/80 (0%) | SSCP and Sanger sequencing |
|  |  |  |  |  | Controls | 0/220 (0%) |  |
| deCid et al., 2010 | Spanish | 51.8/21-76 (ACP)  57.1/9-77 (ICP) | ACP  ICP | All (9) | CP | 6/272 (2.2%) | DDGE, dHPLC, SSCP/HD,  and Sanger sequencing |
|  |  |  |  |  | Controls | 0/186 (0%) |  |
| Midha et al., 2010 | Indian | NA/NA | ICP | All (9) | CP | 1/200 (0.5%) | SSCP/HD and Sanger sequencing |
|  |  |  |  |  | Controls | 0/200 (0%) |  |
| Steiner et al., 2011 | European | 33.0±16.0/NA | ICP | All (9) | CP | 9/252 (3.6%) | DDGE, dHPLC or SSCP/HD |
|  |  |  |  |  | Controls | 4/638 (0.6%) |  |
| Rosendahl et al., 2013 | German | 20.1/1-81 | ICP  HP | p.R74Q, p.R75Q, p.R117H, p.L997F, p.D1152H, p.S1235R | CP | 71/1320 (5.4%) | Melting curve analysis and Sanger sequencing |
|  |  |  |  |  | Controls | 100/3516 (2.8%) |  |
| Masson et al., 2013 | French | NA/<20 | ICP | p.R75Q | CP | 12/506 (2.4%) | dHPLC or HRM analysis |
|  |  |  |  |  | Controls | 25/1028 (2.4%) |  |
| Larusch et al., 2014 | North American | NA/NA | CP | All (9) | CP | 141/1968 (7.2%) | MassARRAY iPLEX Gold assay, TaqMan Gene Expression Assays and Sanger sequencing |
|  |  |  |  |  | Controls | 119/2448 (4.9%) |  |
| Martinez et al., 2014 | French | NA/NA | ICP | p.R75Q | CP | 29/1760 (1.6%) | CF v3 Genotyping Assay, DDGE, dHPLC and Sanger sequencing |
|  |  |  |  |  | Controls | 7/296 (2.4%) |  |
| Muthuswamy et al., 2014 | Indian | NA/NA | NACP | p.R117H | CP | 2/300 (0.7%) | ARMS PCR |
|  |  |  |  |  | Controls | 2/800 (0.25%) |  |
| Schubert et al., 2014 | German | NA/NA | ICP | p.R117H, p.D1152H | CP | 4/194 (2.1%) | CF v3 Genotyping Assay |
|  |  |  |  |  | Controls | 2/260 (0.8%) |  |
| Sisman et al., 2015 | Turkish | 53.2/NA (ACP)  40.4/NA (ICP) | ACP  ICP | p.R117H, p.D1152H | CP | 0/158 (0%) | CF 22Mut and CF 14Mut+Tn strip assay kit |
|  |  |  |  |  | Controls | 0/70 (0%) |  |
| Sofia et al., 2016 | Italian | NA/3–61 | ICP | All (9) | CP | 9/162 (5.6%) | Targeted next-generation sequencing |
|  |  |  |  |  | Controls | 0/100 (0%) |  |
| Philips et al., 2018 | African American | 51.9 ± 10.5/NA | CP | All (9) | CP | 10/464 (2.2%) | TaqMan assays and Sanger sequencing |
|  |  |  |  |  | Controls | 10/476 (2.1%) |  |
| Zou et al., 2018 | Chinese | 40.7±16/6-85 | ACP  ICP  SCP | All (9) | CP | 0/2122 (0%) | Targeted next-generation sequencing |
|  |  |  |  |  | Controls | 0/2392 (0%) |  |
| Iso et al., 2019 | Japanese | NA/≤18 | ICP | All (9) | CP | 0/56 (0%) | Targeted next-generation sequencing |
|  |  |  |  |  | Controls | 0/3000 (0%) |  |
| Chonchubhair et al., 2020 | Irish | 53.2±12.2 | ACP  ICP | p.R117H | CP | 3/252 (1.2%) | TaqMan assay |
|  |  |  |  |  | Controls | 1/334 (0.3%) |  |

MAF, minor allele frequency; *CFTR*^BD^, bicarbonate defective cystic fibrosis transmembrane conductance regulator; CP, chronic pancreatitis; ICP, idiopathic chronic pancreatitis; ACP, alcoholic chronic pancreatitis; NACP, non-alcoholic chronic pancreatitis; SCP, smoking associated chronic pancreatitis; HP, hereditary chronic pancreatitis; NA, not available; dHPLC, denaturing high-performance liquid chromatography; HRM, high-resolution DNA melting analysis; DDGE, denaturing gradient gel electrophoresis; TDGS, two-dimensional gene scanning; SSCP/HD, single-strand conformation polymorphism/heteroduplex; DOVAM-S, detection of virtually all mutations-SSCP; ARMS PCR, amplification refractory mutation system polymerase chain reaction.
