# Supplementary material for: Bicarbonate defective CFTR variants increase risk for chronic pancreatitis: A meta-analysis
Source: PLoS One. 2022 Oct 20;17(10):e0276397. doi: 10.1371/journal.pone.0276397 (PMC9584382; doi:10.1371/journal.pone.0276397)
Supplement: S3 Table — (DOCX) [file pone.0276397.s008.docx]

**S3 Table. Allele frequency of *CFTR^BD^* variants in studies included in the meta-analysis**.

| Study | Cohort | MAF of *CFTR*^BD^ variants | | | | | | | | |
| --- | --- | --- | --- | --- | --- | --- | --- | --- | --- | --- |
|  |  | p.R74Q | p.R75Q | p.R117H | p.R170H | p.L967S | p.L997F | p.D1152H | p.S1235R | p.D1270N |
| Lee et al., 2003 | CP | 0/56 (0%) | 0/56 (0%) | 0/56 (0%) | 0/56 (0%) | 0/56 (0%) | 0/56 (0%) | 0/56 (0%) | 0/56 (0%) | 0/56 (0%) |
|  | Controls | 0/234 (0%) | 0/234 (0%) | 1/234 (0.4%) | 0/234 (0%) | 0/234 (0%) | 0/234 (0%) | 0/234 (0%) | 0/234 (0%) | 0/234 (0%) |
| Fujiki et al., 2004 | CP | NA | NA | 0/130 (0%) | NA | NA | NA | NA | NA | NA |
|  | Controls | NA | NA | 0/324 (0%) | NA | NA | NA | NA | NA | NA |
| Bishop et al., 2005 | CP | 0/74 (0%) | 1/74 (1.4%) | 0/74 (0%) | 0/74 (0%) | 1/74 (1.4%) | 0/74 (0%) | 0/74 (0%) | 1/74 (1.4%) | 0/74 (0%) |
|  | Controls | 0/100 (0%) | 2/100 (2%) | 1/100 (1%) | 0/100 (0%) | 0/100 (0%) | 0/100 (0%) | 0/100 (0%) | 1/100 (1%) | 0/100 (0%) |
| Cohn et al., 2005 | CP | NA | 9/104 (8.7%)* | NA | NA | NA | NA | NA | NA | NA |
|  | Controls | NA | 13/192 (6.8%) | NA | NA | NA | NA | NA | NA | NA |
| Weiss et al., 2005 | CP | 0/134 (0%) | 1/134 (0.7%) | 1/134 (0.7%) | 0/134 (0%) | 0/134 (0%) | 0/134 (0%) | 1/134 (0.7%) | 3/134 (2.2%) | 0/134 (0%) |
|  | Controls | 0/120 (0%) | 0/120 (0%) | 2/120 (1.7%) | 0/120 (0%) | 0/120 (0%) | 1/120 (0.8%) | 0/120 (0%) | 0/120 (0%) | 0/120 (0%) |
| Chang et al., 2007 | CP | 0/146 (0%) | 0/146 (0%) | 0/146 (0%) | 0/146 (0%) | 0/146 (0%) | 0/146 (0%) | 0/146 (0%) | 0/146 (0%) | 0/146 (0%) |
|  | Controls | 0/400 (0%) | 0/400 (0%) | 0/400 (0%) | 0/400 (0%) | 0/400 (0%) | 0/400 (0%) | 0/400 (0%) | 0/400 (0%) | 0/400 (0%) |
| Aoyagi et al., 2009 | CP | 0/80 (0%) | 0/80 (0%) | 0/80 (0%) | 0/80 (0%) | 0/80 (0%) | 0/80 (0%) | 0/80 (0%) | 0/80 (0%) | 0/80 (0%) |
|  | Controls | 0/220 (0%) | 0/220 (0%) | 0/220 (0%) | 0/220 (0%) | 0/220 (0%) | 0/220 (0%) | 0/220 (0%) | 0/220 (0%) | 0/220 (0%) |
| deCid et al., 2010 | CP | 0/272 (0%) | 2/272 (0.7%) | 0/272 (0%) | 1/272 (0.4%) | 0/272 (0%) | 2/272 (0.7%) | 0/272 (0%) | 1/272 (0.4%) | 0/272 (0%) |
|  | Controls | 0/186 (0%) | 0/186 (0%) | 0/186 (0%) | 0/186 (0%) | 0/186 (0%) | 0/186 (0%) | 0/186 (0%) | 0/186 (0%) | 0/186 (0%) |
| Midha et al., 2010 | CP | 0/200 (0%) | 0/200 (0%) | 0/200 (0%) | 0/200 (0%) | 0/200 (0%) | 0/200 (0%) | 0/200 (0%) | 1/200 (0.5%) | 0/200 (0%) |
|  | Controls | 0/200 (0%) | 0/200 (0%) | 0/200 (0%) | 0/200 (0%) | 0/200 (0%) | 0/200 (0%) | 0/200 (0%) | 0/200 (0%) | 0/200 (0%) |
| Steiner et al., 2011 | CP | 0/252 (0%) | 0/252 (0%) | 2/252 (0.8%) | 1/252 (0.4%) | 1/252 (0.4%) | 4/252 (1.6%) | 0/252 (0%) | 1/252 (0.4%) | 0/252 (0%) |
|  | Controls | 0/638 (0%) | 0/638 (0%) | 0/638 (0%) | 1/638 (0.2%) | 0/638 (0%) | 0/638 (0%) | 1/638 (0.2%) | 2/638 (0.3%) | 0/638 (0%) |
| Rosendahl et al., 2013 | CP | 2/1320 (0.2%) | 33/1320 (2.5%)* | 18/1320 (1.4%) | NA | NA | 5/1320 (0.4%) | 3/1320 (0.2%) | 10/1320 (0.8%) | NA |
|  | Controls | 0/3516 (0%) | 61/3516 (1.7%)* | 10/3516 (0.3%) | NA | NA | 6/3516 (0.2%) | 5/3516 (0.1%) | 18/3516 (0.5%) | NA |
| Masson et al., 2013 | CP | NA | 12/506 (2.4%) | NA | NA | NA | NA | NA | NA | NA |
|  | Controls | NA | 25/1028 (2.4%) | NA | NA | NA | NA | NA | NA | NA |
| Larusch et al., 2014 | CP | 3/1968 (0.2%) | 62/1968 (3.2%) | 23/1968 (1.2%) | 3/1968 (0.2%) | 11/1968 (0.6%) | 8/1968 (0.4%) | 4/1968 (0.2%) | 24/1968 (1.2%) | 3/1968 (0.2%) |
|  | Controls | 1/2448 (0.04%) | 76/2448 (3.1%) | 9/2448 (0.4%) | 0/2448 (0%) | 2/2448 (0.1%) | 12/2448 (0.5%) | 0/2448 (0%) | 17/2448 (0.7%) | 2/2448 (0.1%) |
| Martinez et al., 2014 | CP | NA | 29/1760 (1.6%) | NA | NA | NA | NA | NA | NA | NA |
|  | Controls | NA | 7/296 (2.4%) | NA | NA | NA | NA | NA | NA | NA |
| Muthuswamy et al., 2014 | CP | NA | NA | 2/300 (0.7%) | NA | NA | NA | NA | NA | NA |
|  | Controls | NA | NA | 2/800 (0.25%) | NA | NA | NA | NA | NA | NA |
| Schubert et al., 2014 | CP | NA | NA | 3/194 (1.5%) | 1/194 (0.5%) | NA | NA | NA | NA | NA |
|  | Controls | NA | NA | 2/260 (0.8%) | 0/260 (0%) | NA | NA | NA | NA | NA |
| Sisman et al., 2015 | CP | NA | NA | 0/158 (0%) | 0/158 (0%) | NA | NA | NA | NA | NA |
|  | Controls | NA | NA | 0/70 (0%) | 0/70 (0%) | NA | NA | NA | NA | NA |
| Sofia et al., 2016 | CP | 0/162 (0%) | 1/162 (0.6%) | 0/162 (0%) | 0/162 (0%) | 0/162 (0%) | 2/162 (1.2%) | 3/162 (1.9%) | 2/162 (1.2%) | 1/162 (0.6%) |
|  | Controls | 0/100 (0%) | 0/100 (0%) | 0/100 (0%) | 0/100 (0%) | 0/100 (0%) | 0/100 (0%) | 0/100 (0%) | 0/100 (0%) | 0/100 (0%) |
| Philips et al., 2018 | CP | 0/464 (0%) | 2/464 (0.4%) | 0/464 (0%) | 0/464 (0%) | 1/464 (0.2%) | 0/464 (0%) | 0/464 (0%) | 0/464 (0%) | 7/464 (1.5%) |
|  | Controls | 0/476 (0%) | 4/476 (0.8%) | 0/476 (0%) | 0/476 (0%) | 0/476 (0%) | 1/476 (0.2%) | 0/476 (0%) | 0/476 (0%) | 5/476 (1.1%) |
| Zou et al., 2018 | CP | 0/2122 (0%) | 0/2122 (0%) | 0/2122 (0%) | 0/2122 (0%) | 0/2122 (0%) | 0/2122 (0%) | 0/2122 (0%) | 0/2122 (0%) | 0/2122 (0%) |
|  | Controls | 0/2392 (0%) | 0/2392 (0%) | 0/2392 (0%) | 0/2392 (0%) | 0/2392 (0%) | 0/2392 (0%) | 0/2392 (0%) | 0/2392 (0%) | 0/2392 (0%) |
| Iso et al., 2019 | CP | 0/56 (0%) | 0/56 (0%) | 0/56 (0%) | 0/56 (0%) | 0/56 (0%) | 0/56 (0%) | 0/56 (0%) | 0/56 (0%) | 0/56 (0%) |
|  | Controls | 0/3000 (0%) | 0/3000 (0%) | 0/3000 (0%) | 0/3000 (0%) | 0/3000 (0%) | 0/3000 (0%) | 0/3000 (0%) | 0/3000 (0%) | 0/3000 (0%) |
| Chonchubhair et al., 2020 | CP | NA | NA | 3/252 (1.2%) | NA | NA | NA | NA | NA | NA |
|  | Controls | NA | NA | 1/334 (0.3%) | NA | NA | NA | NA | NA | NA |

With the exception of p.R75Q, all variants were found in the heterozygous state. * indicates cohorts where homozygous p.R75Q was present (2 patients and 1 control in Rosendahl et al.; 1 control in Cohn et al.)

MAF, minor allele frequency; *CFTR*^BD^, bicarbonate defective cystic fibrosis transmembrane conductance regulator; CP, chronic pancreatitis; NA, not available.
